# Evaluation of a deformable image registration algorithm for image‐guided thermal ablation of liver tumors on clinically acquired MR‐temperature maps

**DOI:** 10.1002/mp.17526

**Published:** 2024-11-23

**Authors:** Valéry Ozenne, Pierre Bour, Thibaut Faller, Manon Desclides, Baudouin Denis de Senneville, Osman Öcal, Sergio Lentini, Max Seidensticker, Olaf Dietrich, Bruno Quesson

**Affiliations:** ^1^ CNRS, CRMSB, UMR 5536, IHU Liryc University of Bordeaux Bordeaux France; ^2^ Certis Therapeutics Pessac France; ^3^ University of Bordeaux, CNRS, INRIA, Bordeaux INP, IMB UMR 5251 Talence France; ^4^ Department of Radiology University Hospital LMU Munich Munich Germany

**Keywords:** deformable image registration, dosimetry, echo planar imaging, optical flow, respiratory motion, thermo‐ablation, thermometry

## Abstract

**Background:**

Quantitative real‐time MRI‐based temperature mapping techniques are hampered by abdominal motion. Intrascan motion can be reduced by rapid acquisition sequences such as 2D echo planar imaging (EPI), and inter‐scan organ displacement can be compensated by image processing such as optical flow (OF) algorithms. However, motion field estimation can be seriously affected by local variation of signal intensity on magnitude images inherent to tissue heating, potentially leading to erroneous temperature estimates.

**Purpose:**

This study aims to characterize, in the context of clinical MRI‐guided microwave ablation (MWA), a novel deformable image registration (DIR) algorithm that enhances the generation of thermal maps aligned to a reference position, a critical step for calculating cumulative thermal dose and, consequently, for the real‐time evaluation of interventional procedure progress.

**Methods:**

A retrospective image analysis was performed on 11 patients that underwent MWA of a liver tumor (primary or metastasis). Ablation duration was set to 9 ± 2 min with a 14‐gauge large antenna. A stack of 13–20 contiguous slices was acquired dynamically (350 repetitions) at 1.5T using a single‐shot EPI sequence. Evaluation was first performed on motion‐free datasets (5 gated acquisitions using a cushion positioned in the patient abdomen) then with ones with motion (8 fixed‐frequency acquisitions at 0.5 Hz). Temperature, thermal dose and lesion size were computed using three workflows: (i) standard phase subtraction (gold standard), (ii) conventional OF motion compensation, (iii) PCA‐based OF motion compensation. The impact of flow field, temperature and lesion volume estimation were compared using averaged endpoint error (AEE), NRMSE and bland Altman plot, respectively.

**Results:**

Intensity signal decreases (close to 50%) were observed in the vicinity of the probe during MW energy delivery. Both motion correction algorithms reduce the NRMSE of magnitude images throughout the acquisition (*p* < 0.005) and achieve similar results between them. *Gated acquisition results*. Conventional OF produced erroneous vector fields compared to the PCA‐based OF, leading to higher maximal EE (3 mm vs. 1 mm) and temperature errors up to 15°C–20°C. PCA‐based OF algorithm significantly reduces the NRMSE of temperature (*p* < 0.005). The conventional OF method underestimated the final size of lesions with a bias of 0.93 cm^3^ while the PCA‐based OF reported a bias of 0.5 cm^3^. *Fixed frequency acquisition results*. The temperature estimation without motion correction led to strong fluctuations or loss of temperature measurement while the proposed PCA‐based OF recovered both a stable and precise measurement with null bias.

**Conclusion:**

The deformable image registration algorithm is less sensitive to local variations of the signal. Volumetric temperature imaging without gating (20 slices/2 s) could be performed with the same accuracy, and offer trade‐offs in acquisition time or volume coverage. Such a strategy is expected to increase procedure safety by monitoring large volumes more rapidly for MR‐guided thermotherapy on mobile organs.

## 1 INTRODUCTION

Percutaneous thermal ablation of oligo metastatic disease or early‐stage hepatocellular carcinoma (HCC) is an established therapy since guidelines on many disease modalities recommend thermoablation as a first‐line treatment according to the number and size of the lesion.^1^, ^2^, ^3^, ^4^, ^5^, ^6^ Interventional thermoablation procedures aim at destroying pathological tissues via a localized energy deposit. Ideally, the resulting ablated zone is aimed to cover the lesion borders with additional margins while avoiding unwanted damage to the surrounding tissues. The size of HCC varies a lot (from <1 to >10 cm) but standard treatment with a single probe under imaging guidance assumes a maximum size of 3 cm^7^ In the absence of precise temperature mapping during the procedure, empirical energy delivery (power and emission time) are selected by the clinician according to recommendations of the vendor (typically based on ex vivo experiments). As a result, personalized treatment remains difficult and the prediction of the resulting ablated zone remains imprecise since, in vivo, the size of the ablation zone is affected by perfusion, heat‐sink effects due to large vessels, and individual tissue composition. Thermal ablation procedures are associated with a 6%−12% local recurrence due to incomplete lesion coverage despite contrast‐enhanced control images acquired at the end of the procedures.^6^, ^8^ Real time quantitative monitoring of thermal energy deposition appears necessary to improve therapeutic procedures and to avoid heat‐induced complications in surrounding healthy tissues. MRI has the ability to non‐invasively monitor local temperature changes during the thermal therapies using quantitative real time temperature mapping techniques.^9^, ^10^, ^11^ The linear dependence of proton resonance frequency (PRF) shift on temperature is the basis of MR thermal monitoring.^12^ The standard method is based on phase mapping techniques using gradient echo imaging and measures the relative change in temperature throughout the acquisition in each voxel of the image. Knowing the local tissue temperature, it is then possible to predict cell death using the thermal dose calculation in each voxel. In particular, real time monitoring of the thermal dose distribution at the target region can be used to determine (and minimize) the required duration of the thermal ablation, whereas monitoring of the temperature distribution in adjacent risk structures is required to be able to interrupt the ablation if predefined temperature thresholds should be exceeded.

Nevertheless, respiratory motion causes significant challenges in abdominal organ imaging.^13^ Apnea cannot be an option for a typical treatment with duration of several minutes and synchronization of the image acquisition with the physiological motion is currently used.^13^, ^14^ This approach has been applied recently with FLASH sequences during MWA on patients.^10^, ^15^ However, these approaches remain relatively slow with an update time equal to the typical duration of the physiological motion (about 6−7.5 s for one slice with FLASH sequence), leading to limited spatial coverage and temporal resolution of the thermometry. As a result, the characterization of the spatial extent of the ablation and precise computation of the accumulated thermal dose from temperature maps is limited.

An alternative three‐step approach is to acquire the image continuously using very fast acquisition sequence (<100 ms per slice), such as single‐shot EPI,^16^ to eliminate intra‐scan motion. Inter‐scan motion, corresponding to the change in organ position (∼ 4–5 voxels or 9–12 mm apart) between two successive scans, must be corrected to follow the temporal evolution of the temperature in each voxel throughout the procedure. For this purpose, deformable image registration (DIR) methods^17^ such as optical flow (OF) algorithms exploit the conservation of intensity between successive images and estimate a displacement field per voxel that reflects the motion of each region of the image. The estimated motion can then be applied to phase images in a two‐step procedure: (i) Each phase image is registered to a fixed reference position based on the estimated motion field. (ii) Assuming a linear relationship between periodic organ displacement and phase variation, a phase correction also referred to as correction of magnetic susceptibility artifacts^18^ is performed to compute the temperature.

In such context, local intensity changes during MWA (due to changes in tissue MR properties with heating) may be interpreted by the algorithm as a local motion and lead to erroneous estimation of local vector fields and final temperature estimates. Errors up to 10°C have been reported in static agar gel experiments^19^ using this strategy. A novel motion compensation algorithm^20^ was proposed to constrain the computation of motion fields during the heating period and to deliver non‐compromised temperature maps. This algorithm has been evaluated in silico and on preclinical data^21^ but not in the context of clinical ablation.

The purpose of this study is to evaluate a novel DIR algorithm that enhances the generation of thermal maps aligned to a reference position, a critical step for calculating cumulative thermal dose and, consequently, for real‐time control and evaluation of interventional procedure progress as explained above. The method was retrospectively evaluated on datasets from patient that underwent MWA. The proposed approach was first evaluated on motion‐free datasets (gated acquisitions) to compare the robustness of the proposed OF method with the original method and to ensure the presence of a gold standard reference. Then, to demonstrate the feasibility and advantages of the proposed method under actual treatment conditions, the method was applied to motion datasets (fixed‐frequency acquisitions).

## 2 METHODS

### Study population

Data were derived from patients who underwent microwave ablation (MWA) of primary or secondary liver tumors under MRI monitoring at the University Hospital of Munich within a prospective trial (Clinical Trial Register Number: DRKS00028515) between December 2020 and July 2023. The additional post hoc analysis was approved by the ethics committee of LMU Munich, and informed consent was waived due to the retrospective nature of the study. A total of 11 patients (36% female, 64% male, age 65 ± 9 years) with 13 lesions were included in the analysis. Reablation after needle repositioning was performed in two patients (G#4–G#5) and (FF#7–FF#8).

### Ablation procedure

During the procedure, each patient was under general anesthesia with the permanent care of medical staff. An AveCure microwave system (MedWave, San Diego, USA) was used to perform the ablation using a 14‐gauge large antenna inserted percutaneously under MRI guidance. The device was connected to a generator located outside the Faraday cage using a shielded cable provided by the manufacturer. Ablation duration was set to 9 ± 2 min with a target temperature of 120°C (based on the lesion size, the recommendations of the vendor, and previous experience with the MWA system) and a delay of 30 s was observed before starting the energy deposition. The size of the ablation zone (on day one after ablation) is typically smaller than expected from vendor recommendation. To overcome too‐small ablation zones, the ablation time is usually increased in order to reach sufficiently large ablation volumes. With the chosen parameters, ablation volumes were clinically adequate in the presented cases.

### Thermometry acquisition

A stack of slices was acquired dynamically on a 1.5T MRI scanner (Magnetom Aera, Siemens Healthineers, Erlangen, Germany) using a single‐shot gradient‐echo echo planar imaging (EPI) sequence: matrix size = 128 × 128, slice thickness = 3 mm, no slice gap, TE = 18 ms, TR = 2000 ms, FA = 90°, pixel bandwidth = 1445 Hz/pixel, phase encoded direction = left‐right, GRAPPA acceleration factor = 2, 6/8 partial Fourier, echo spacing = 0.78 ms, echo train length 47. Parameters varying between different acquisitions (total acquisition time, number of slices, number of repetitions, field of view, spatial resolution …) are listed in Table 1. To avoid aliasing of the patient's arms, two saturation bands were positioned on each side of the patient. Two strategies were chosen. MRI acquisitions of five microwave treatments were performed under respiratory gating during the exhalation phase using a cushion positioned in the abdomen of the patient. To freeze the motion, gated acquisitions limit the acquisition window to the most stable part of respiration (i.e., approximately 1 s in expiration), thereby reducing spatial coverage. The maximum image update rate is then equal to the respiratory period (approximately 0.2 Hz). Any small shift in the respiratory cycle between the reference image and the current image will introduce both a positional error in the voxels located on the image and a difference in magnetic susceptibility. This can lead to significant temperature bias. Eight acquisitions were also performed without respiratory gating. In this case, the acquisitions are triggered every 2 s (or at a fixed frequency of 0.5 Hz), which is about twice as fast as the respiratory rate of patients under general anesthesia. The stack of slices was acquired in paracoronal or parasagittal to minimize through‐plane motion and to locate the microwave antenna in the central slice of the stack. The MRI DICOM phase and magnitude data were transmitted in real time to a workstation to calculate temperature maps using the software “Certis Solution” version 1.2.0 (Certis Therapeutics, Pessac, France) and displayed in the console room with a delay and a frame rate of approximately 2 s. The frame rate was limited by the acquisition rate of the sequence listed in Table 1. A different image and temperature reconstruction framework is used in this study.

**TABLE 1:** Acquisition parameters. Five acquisitions (named “G”) were performed under respiratory gating while eight acquisitions (named “FF”) were performed without gating with a fixed frequency of 0.5 Hz.

PatientNb	TotalAcqTime (min‐sec)	FrameRate (ms)	Repetitions	SlicesNb	FOVx (mm)	FOVy (mm)	Resx (mm)	Resy (mm)
G 1	12m 49s	3528 +/− 88	219	20	320	320	2.5	2.5
G 2	11m 5s	5001 +/− 78	134	13	320	320	2.5	2.5
G 3	12m 20s	3334 +/− 61	223	13	280	280	2.2	2.2
G 4	15m 25s	5143 +/− 450	181	13	300	300	2.3	2.3
G 5	10m 55s	5002 +/− 66	132	13	300	300	2.3	2.3
FF 1	14m 58s	2000 +/− 0	450	13	320	320	2.5	2.5
FF 2	9m 18s	2000 +/− 0	280	12	340	340	2.7	2.7
FF 3	12m 02s	2070 +/− 0	350	13	230	230	1.8	1.8
FF 4	7m 58s	2000 +/− 0	240	20	270	270	2.1	2.1
FF 5	10m 38s	2000 +/− 0	320	13	260	260	2	2
FF 6	11min38	2000 +/− 0	350	16	230	230	1.8	1.8
FF 7	12m 38s	2000 +/− 0	380	20	250	250	2	2
FF 8	8m 18s	2000 +/− 0	250	20	230	230	1.8	1.8

### Image reconstruction

The spine coil integrated into the MRI bed and a loop coil positioned on the abdomen and surrounding the insertion point of the device were used for data acquisition (13 receiver channels for image reconstruction). The raw data files were converted into ISMRM Raw Data format and reconstructed using the Gadgetron framework.^22^ Initial steps included regridding, EPI ghost‐Nyquist correction and coil compression. The Gadgetron implementation of GRAPPA and partial Fourier (PF) reconstruction were then applied to correct for phase‐encoding undersampling in image reconstruction.

### Deformable image registration

**FIGURE 1:**
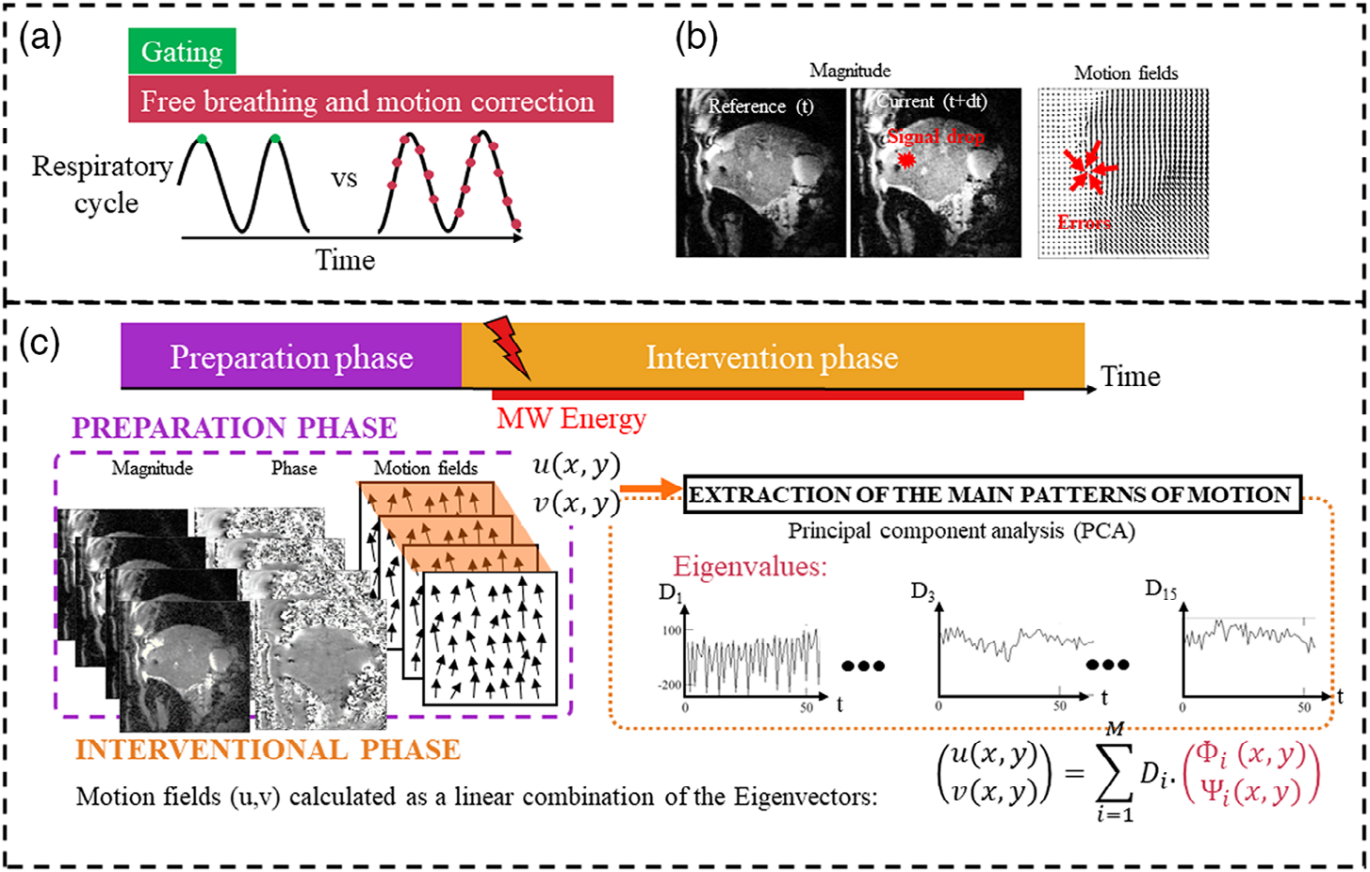
Schematic diagram of the problematic and proposed implementation. (a) Triggering method. (b) Magnitude signal drop impact motion field estimation. (c) The 2D estimated motion fields (u, v) are collected in the preparation phase. A PCA is then performed on the motion fields of the learning step. During the interventional procedure, for each new incoming image, the 2D motion is estimated as a linear combination of the previously computed eigenvectors. The displacements represented by the arrows are illustrations and are not displayed to scale.

For each image in the time series, the displacement field (u,v) was estimated from the magnitude images and used to register both magnitude and phase images to a fixed reference position. The reference position is defined as the median position estimated from the different positions observed over the first 10 stacks of slices acquired. In practice, it will find a location near the peak of the breathing cycle for gated acquisition (dark green dots in Figure 1a) while it will find a location in the middle of the respirator cycle for fixed frequency acquisition (dark red dots in Figure 1a). This solution minimizes the displacement when estimating and applying the vector field.

Since OF algorithms rely on the local conservation of the intensity, the drop in signal intensity induced by heating and changes in tissue properties may introduce errors in the estimated motion (u, v). Therefore, two different OF algorithms have been evaluated:

1. The conventional OF using a Horn and Schunck (H&S) implementation^17^ computes vector fields representing displacement between each new magnitude image and the image at the reference position.
2. The PCA‐based OF method introduced in^20^ (see Figure 1) identifies spatial and temporal consistencies in the motion of the observed region through preparative learning covering several breathing cycles. This enables, during hyperthermia, the elimination of mis‐registration inherent to tissue heating. The method requires a preparative learning step covering several breathing cycles before starting the ablative procedure (∼15 frames). The 2D estimated motion fields (u, v) are collected together with the registered phase images using the conventional OF method^17^ since local variations of intensity are unexpected. A PCA is then performed on the motion fields of the learning step to extract eigenvector maps and eigenvalues. During the interventional procedure (from the sixteenth frame to the end), for each new incoming image, the 2D motion is estimated as a linear combination of the previously computed eigenvectors.

Conventional OF and PCA‐based OF methods were both used to register magnitude/phase images in order to evaluate the impact of the algorithms on MR thermometry, dosimetry (computed using the equivalent minutes at 43°C), and lesion volume estimation.

### 2.1 Correction of respiratory‐induced susceptibility artifacts

Correction of respiration‐induced susceptibility artifacts was then performed by parameterizing the phase using a PCA on a pixel‐by‐pixel basis. Details on this method can be found in Maclair et al^23^ or refs. [24, 25]. The approach is divided into two steps: a learning phase during which the influence of the displacement of the phase susceptibility is estimated using a model parameterization, and an intervention step during which the phase correction is estimated based on the actual motion state and subtracted from the current temperature image to remove the motion‐induced susceptibility artifacts. It should be noted that the learning phase (from the first frame to the fifteenth frame) and the intervention phase (from the sixteenth frame to the end) can be defined in the same way as in the previous step concerning image registration. In the learning phase, a PCA was applied to a collection of motion fields to obtain motion descriptors. A first‐order variation of local phase changes due to motion is then considered and can be written as a linear combination of motion descriptors and a set of parameterized magnetic field models. During the intervention, the largest PCA‐based motion descriptors were estimated from the current motion field and the background phase $\varphi_{\mathrm{ref}}$ was computed for each incoming acquisition.

For clarity, it should be pointed out that the methodology uses two methods based on Principal Component Analysis. The first, referred to throughout the article as “PCA‐based,” indicates that the motion fields during the intervention phase will be a linear combination of those observed during the preparatory phase. The second, called “Correction of respiratory‐induced susceptibility artifacts” indicates that the background phase $\varphi_{\mathrm{ref}}$ is a model parametrization. The second was thus applied to the motion fields of the two OF algorithms: “Conventional” and “PCA‐based.”

### 2.2 Temperature calculation

Temperature calculation was performed using the PRF method that computes temperature change ΔT from the difference between a given phase image $\varphi_{t}$ acquired during treatment and a reference phase image $\varphi_{\mathrm{ref}}$ acquired prior to heating.^26^

$$\Delta T=\left(\varphi_{t}-\varphi_{\mathrm{ref}}\right)\;.{\left(\gamma .\sigma .\mathrm{TE}.B_{0.}\right)}^{-1}$$

where γ is the gyromagnetic ratio (≈42.58 MHz T−1), σ = −0.0094 ppm·°C−1 is the PRF temperature coefficient, $B_{0.}$ is the magnetic field strength (1.5 T here) and TE is the echo time.

The temperature change estimation ΔT was carried out using the phase subtraction described in Equation (1) but with three different reference phases:

1. Gold standard method: with fixed reference phase image $\varphi_{\mathrm{ref}}$
2. Conventional OF: with the computed background phase $\varphi_{\mathrm{ref}}$ computed from motion fields estimated with the conventional OF method.
3. PCA‐based OF: with the computed background phase $\varphi_{\mathrm{ref}}$ computed from motion fields estimated with the PCA‐based OF method.

Spatial‐temporal drift correction and temporal filtering using a first‐order low‐pass Butterworth filter with a cutoff frequency of 0.14 Hz were finally applied based on this initial implementation.^25^

### 2.3 Thermal dose and lesion volume estimation

The cumulative thermal dose (TD) also described as the cumulative equivalent minutes (CEM) was computed from the temperature images using the Sapareto equation.^27^  The latter establishes an empirical relationship between the absolute temperature, the exposure time, and cell death. The lesion volume was estimated by taking an equivalent dose of 240 min at 43°C (CEM_43_), as it is the theoretical threshold of cell death. The initial temperature was set to 37°C for each patient.

### 2.4 Data analysis

The data analysis has two objectives: first, to compare the robustness of the proposed OF method with the original method. Second, to demonstrate the feasibility and advantages of the proposed method under actual treatment conditions.

For the first objective, image data sets from five treatments performed under gated MRI acquisitions are used, assuming an absence of residual inter‐scan motion. Under these conditions, the gold standard temperature map ΔT is calculated by simple phase subtraction without DIR. Temperature maps were then calculated after DIR using conventional OF and PCA‐based OF methods. Differences in motion field, temperature, and lesion volume size estimation were compared for the three methods using the metrics described below.

For the second objective, image data sets from eight treatments were performed without MRI gating. Under these conditions, the standard subtraction (formerly the gold standard) method was not relevant due to the presence of motion but was calculated and displayed for information purposes. Nevertheless, to a certain extent, the standard subtraction was considered, in some voxels, as a good indicator of temperature: for some cases, the limited motion (due to general anesthesia) and the large and long ablation (the spatial gradient temperature between voxels was relatively low) helped to estimate the “true” temperature. The two different OF algorithms and the resulting metrics were then compared qualitatively.

In gated acquisitions, the acquisition frame rate was considered equal to the patient's respiratory rate imposed by the ventilator. The exact frame update was extracted from the timestamp of the kspace data. To characterize the regularity of the respiratory motion and image similarity, the Intercorrelation coefficient was computed between the current magnitude image and the reference magnitude image selected from the first 10 images and located in the middle of the respiratory cycle. The metric is computed on the whole image and averaged overall slices.

$$\mathrm{Corr}\;\left(I,J\right)=\frac{\sum \left(I-\hat{I}\right)\left(J-\hat{J}\right)}{\sqrt{\sum {\left(I-\hat{I}\right)}^{2}\sum {\left(J-\hat{J}\right)}^{2}}}$$

where (I, J) are the current magnitude and reference magnitude images.

Motion correction performance and image similarity quality was then evaluated with the three methods by computing over all dynamic acquisitions the normalized root mean square error (NRMSE) between the current and the reference stack of images:

$$\mathrm{NRMSE}\;\left(I,J\right)=\sqrt{\frac{\sum_{i=1}^{N}{\left(I_{i}-J_{i}\right)}^{2}}{N}}$$

where (I, J) are the current magnitude and reference magnitude stack of images and *N* the total number of voxels within the stack of images.

The potential artifact on temperature images introduced by erroneous motion correction due to signal intensity variation on magnitude images was quantified by calculating the resulting bias in image registration. To do this, the averaged endpoint error (AEE) was calculated for each repetition over a ROI of 19 × 19 voxels centered on the ablated area. The metric is calculated on the extent of the ablation, which corresponds to a minimum of 4 slices and up to 20 slices depending on the patient and acquisition setup. As the SNR reduction is very localized in the vicinity of the needle, only voxels with temperature higher than 10°C during the course of the ablation were included in the spatial averaging. The statistical analysis in the whisker diagram was then computed over 100 dynamic acquisitions taken during the heating period.

$$\begin{array}{cc} \mathrm{EE}\;(\mathrm{rep}) & =\sqrt{{\left(u-u_{\mathrm{ref}}\right)}^{2}+{\left(v-v_{\mathrm{ref}}\right)}^{2}}\;\;\mathrm{and}\;\mathrm{AEE}\left(\mathrm{rep}\right)\\ & =\frac{1}{N}\sum_{i=1}^{N}EE_{i\;\left(\mathrm{rep}\right)} \end{array}$$

where (u, v) and ($u_{\mathrm{ref}}$, $v_{\mathrm{ref}}$) are the estimated and the reference motion, respectively. ($u_{\mathrm{ref}}$, $v_{\mathrm{ref}}$) is the motion estimated using the gold standard method while (u, v) is the motion computed individually with the conventional OF and the PCA‐based OF. And *i* are the voxel location within the volume where the temperature is higher than 10°C during the course of the ablation.

Thermometry performance was evaluated for each motion compensation algorithm for computing:

1. The NRMSE and the absolute temperature error. The evaluation was carried out on a 19 × 19 ROI centered on the ablated area. Such metrics were used to measure and compare the temperature bias between the gold standard and the proposed DIR methods. The statistical analysis in the whisker diagram was computed over 100 dynamic acquisitions taken during the heating period.

Dosimetry and lesion volume estimation performance were evaluated for each motion compensation algorithm by computing:

1. The accumulated thermal dose (CEM_43_), the time to reach the accumulated thermal dose threshold (in dynamic acquisition), the difference in time to reach the accumulated thermal dose threshold. These metrics were used to measure and compare the potential bias between the gold standard and the proposed motion compensation algorithms.
2. The lesion volume estimated in cm^3^ over 10 consecutive dynamic acquisitions. The bias in volume estimation between the gold standard and the proposed DIR methods is then assessed using the Bland–Altman plot.

## 3 RESULTS

Figure S1 shows the influence of respiration and/or liver motion on image similarity metrics in three different cases using (i) a temporal plot of intensity profile through the liver and (ii) the intercorrelation coefficient of magnitude images through the acquisition. The first case (G#1) is a respiratory‐gated acquisition, no significant difference is observed in the intensity profile, and the intercorrelation coefficient is higher than 0.95 indicating a high image similarity. A slight decrease is observed at the end of the procedure. In the second case (FF#2) in Figure S1b, the acquisition was carried out without respiratory gating at a fixed frequency of 0.5 Hz. Regular small oscillations, linked to breathing, were visible both on the intensity profile and on the intercorrelation coefficient, which remained above 0.9. The last case (FF#8) in Figure S1c is also carried out without respiratory gating at a fixed frequency of 0.5 Hz. Small oscillations were observed at the beginning of the procedure on both panels followed by a sudden change around dynamic acquisition #40 (*t* = 80 s) linked to a sudden contraction of the organ during ablation. The intercorrelation coefficient then falls from 0.9 to 0.8 and gradually rises back to 0.85 at the end of the procedure. This last case was therefore excluded. Figure S2 compares image similarity quality through the acquisition using the intercorrelation coefficient of magnitude images for both gated and non‐gated acquisitions. All gated acquisitions had a score higher than 0.95, while the fixed frequency acquisitions have a score lower but always higher than 0.9.

### 3.1 Gated acquisition results

**FIGURE 2:**
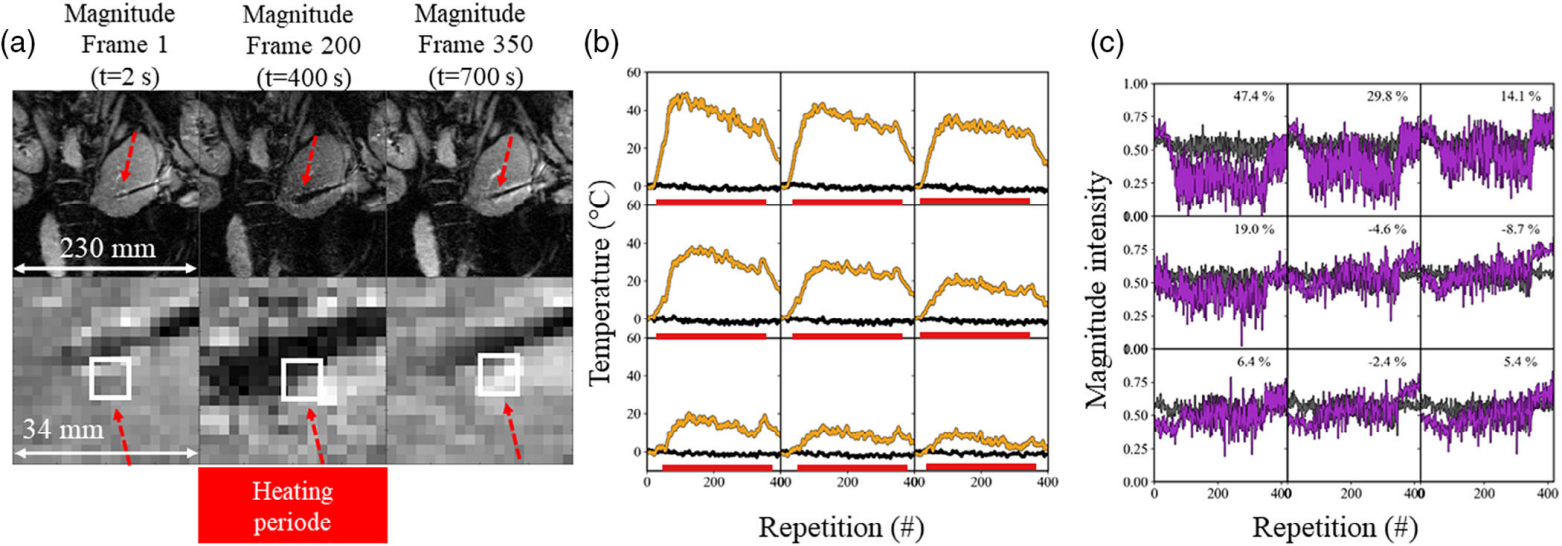
Representative case of large and local intensity variations during ablation. (a) The magnitude image and a zoomed view close to the tip of the needle (indicated by red arrows) are shown in one slice at dynamic acquisition #1 (*t* = 2 s), #200 (*t* = 400 s), and #350 (*t* = 700 s). Temperature (b) and signal intensity (c) evolution as a function of dynamic acquisition are shown in a 3 × 3 kernel of voxels in yellow and purple respectively. The red line indicates the heating period. The location of the kernel is indicated by a white box in panel A. The black lines are another 3 × 3 kernel of voxel located far from the needle extremity. The maximal signal decrease of the magnitude signal is indicated in % at the corner of the voxel.

Figure 2a shows a representative case (FF #6) of MW ablation with magnitude images at *t* = 0 s (before energy delivery) and *t* = 400 s (during the heating period) and *t* = 700 s (during the cooling period). A black hypo intense signal appears around the needle during ablation. It has disappeared toward the end of the ablation and has been replaced by a smaller white hyper intense signal. Figure 2b,c show temporal measurements of temperature and magnitude as a function of time in a 3 × 3 kernel of voxels near the ablation spot. An approximate maximum temperature change of +50°C was observed. At the end of the procedure, a decrease of temperature is observed although the acquisition was stopped before temperature returned to the baseline. In this experiment, a maximal signal decrease of 47% of original magnitude signal intensity was observed.

**FIGURE 3:**
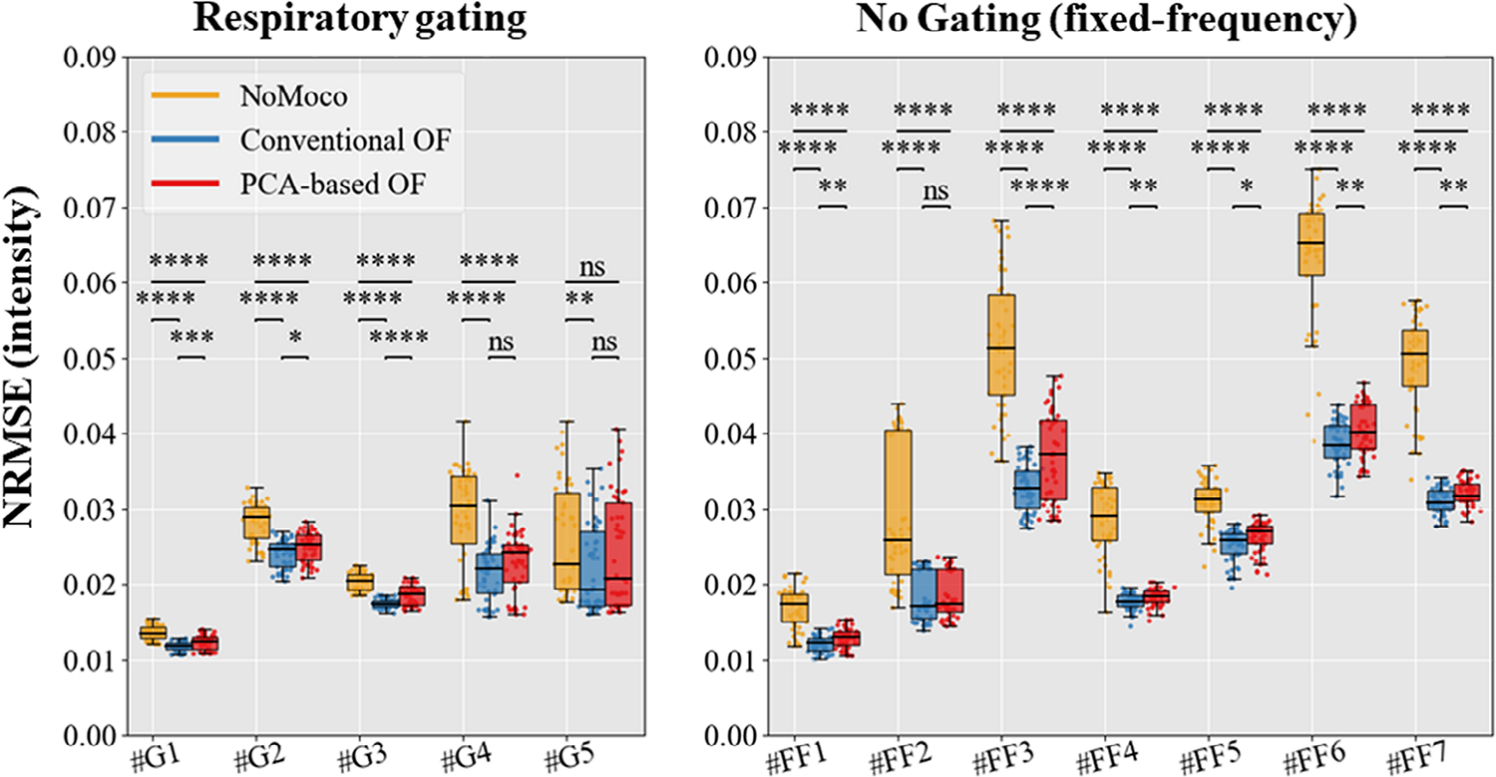
Comparison of image similarity quality with or without DIR for respiratory‐gated (left) and non‐gated (right) acquisition. The box and whisker of the NRMSE between the image intensity of the current frame and the reference frame are plotted for the conventional OF in blue, the PCA‐based OF in red, and without any motion correction in yellow. DIR, deformable image registration; NRMSE, normalized root mean square error; OF, optical flow.

Figure 3 compares the NRMSE of magnitude images through the acquisition with and without DIR for both gated and non‐gated acquisitions. For gated acquisition, the use of DIR has a moderate impact in NRMSE even if a significant decrease is noticeable for the cases G#1 to G#4. For non‐gated acquisition, the NRMSE calculated without motion correction (in gold) has both a wider distribution and a higher value than for the gated acquisition. The addition of OF approaches drastically reduces both the width of the distribution and the median for all acquisitions. Lastly, no major differences were observed between the two algorithms *Conventional OF* (in blue) and *PCA‐based OF* (in red) with the exception of the case FF#3 which is slightly outperformed using the *Conventional OF* approaches.

**FIGURE 4:**
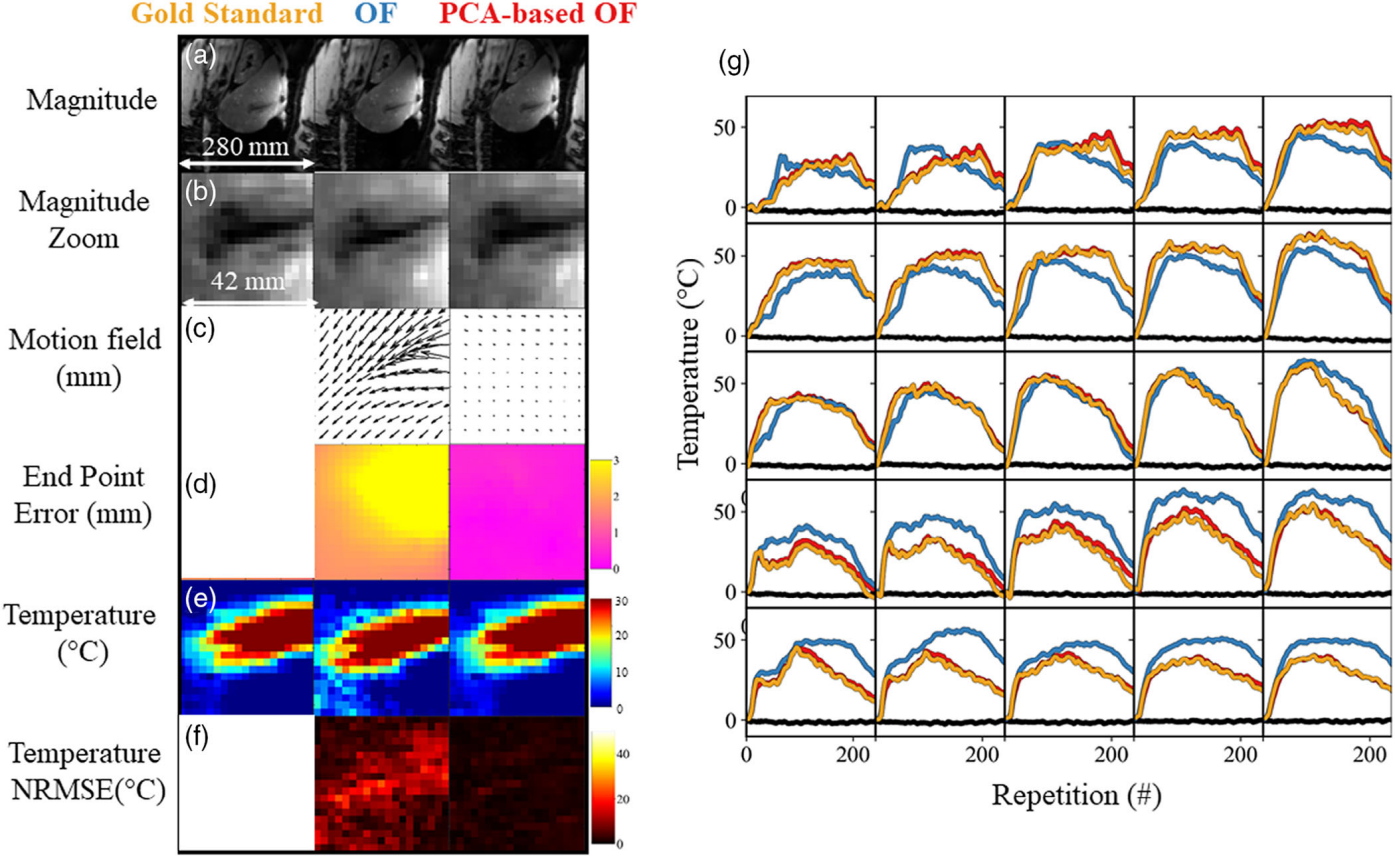
Case study of a gated acquisition for comparing the thermometry performance with the proposed methods. Left panel: The first column corresponds to the *Gold standard (in gold)*, the second to the *Conventional OF* (in blue) and the last one to the *PCA‐based OF (in red)*. Magnitude image (a) and a zoomed view (b) are shown close to the tip of the needle. Estimated motion field (c) after regridding (for a correct visualization) and End Point Error (d) are then shown. Motion fields are assumed to be equal to zero between consecutive images due to gating. Lastly, temperature and temperature error are shown. Temperature errors. Right panel: Temperature evolution as a function of dynamic acquisition is shown in a 5 × 5 kernel of voxels using the color code introduced above. The black lines are another 5 × 5 kernel of voxel located far from the needle extremity. OF, optical flow.

**FIGURE 5:**
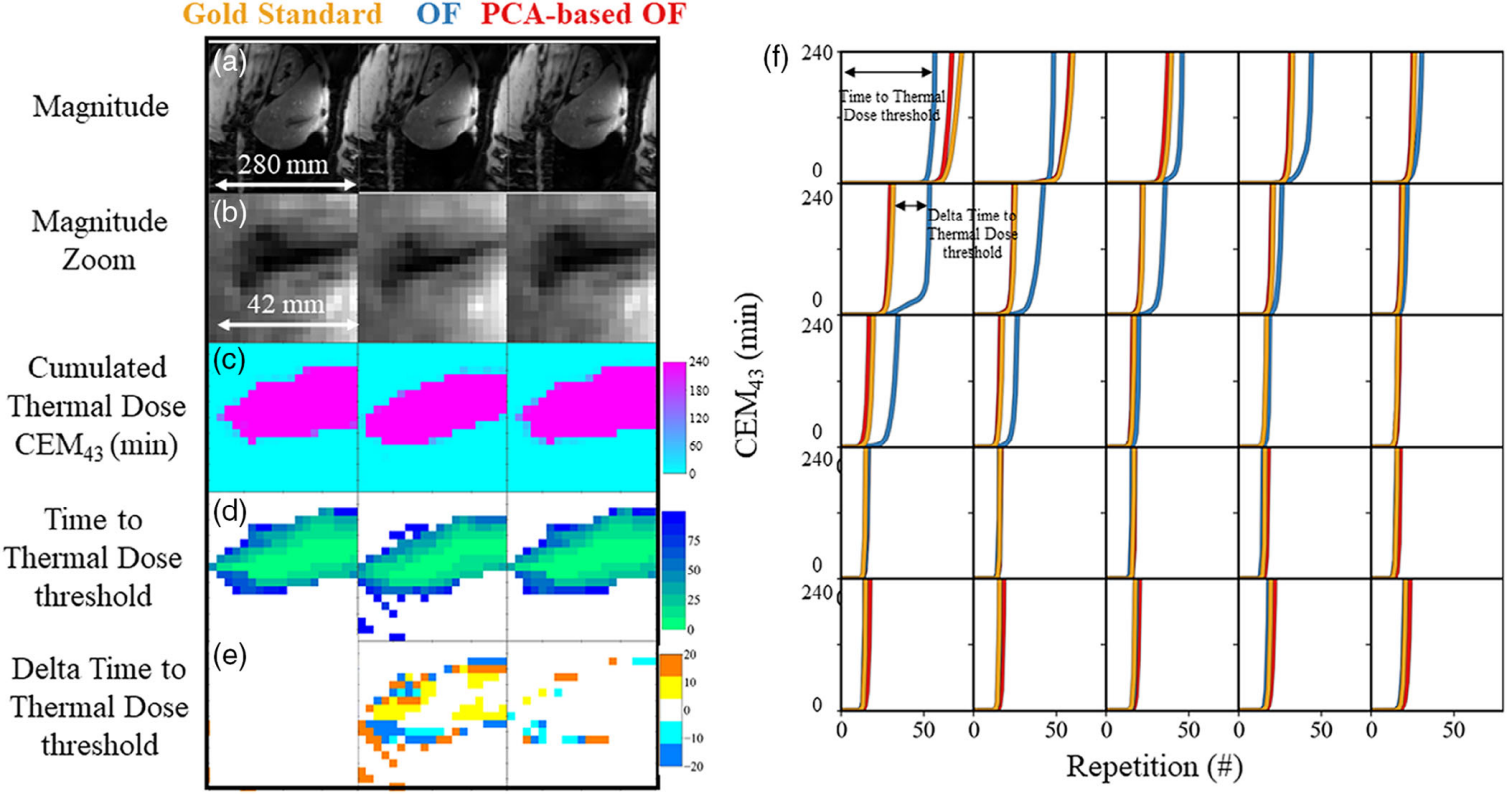
Case study of a gated acquisition for comparing the dosimetry performance with the proposed methods. Left panel: The first column corresponds to the *Gold standard (in gold)*, the second to the *Conventional OF* (in blue), and the last one to the *PCA‐based OF (in red)*. Magnitude images (a) and a zoomed view (b) are shown close to the tip of the needle. CEM_43_ (c), the time to reach the thermal dose threshold (240 min at CEM_43_) (d), and the difference in time to reach the accumulated thermal dose threshold (e) are then shown. Right panel: Cumulated thermal dose evolution as a function of dynamic acquisition is shown in a 5 × 5 kernel of voxels using the color code introduced above. The *x*‐axis has been shortened for visibility. CEM, cumulative equivalent minutes; OF, optical flow.

Figure 4 compares the thermometry performance with the proposed OF algorithms on a respiratory‐gated acquisition (G#3). The gold standard method is a simple phase subtraction with a fixed reference phase image. This assumption remains valid only if there is no motion during the acquisition, which has been verified above. The first and second rows (Figure 4a,b) show the magnitude images and a zoomed view in which the liver and MW needle are clearly visible. Although no motion is expected to be present, the conventional OF estimated, at dynamic acquisition #100, a higher vector field (Figure 4c) than the PCA‐based OF. The maximum EE (Figure 4d) was 2.67 mm with OF and 0.98 mm with PCA‐based OF. At first glance, the temperature map (Figure 4e) displays a similar pattern, but some variations can be noticed. Using the conventional OF method, the temperature NRMSE (Figure 4f) was found higher than 10°C in a large part of the voxel at the vicinity of the needle. As a result, the maximum temperature reached is wrong and the estimation of coagulation necrosis via the thermal dose will be strongly biased. Such a temperature difference is therefore important from a clinical point of view for the safety and accuracy of the procedure. In contrast, using the PCA‐based method, the temperature NRMSE was found below 2°C in most voxels. A few voxels located on the needle are impacted by the needle artifact itself. Temperature evolution in time in a ROI of 5 × 5 pixels was plotted in the right panel (Figure 4g) and illustrates the bias introduced by both tested methods versus the gold standard (in gold). Temperature errors up to 15°C−20°C are visible in 2 voxels. This bias can either lead to over or underestimation of the measurement in the same voxel (top left) through the acquisition or to underestimation of the measurement (at the center). To illustrate the reproducibility and reliability of the method, one additional case (G#5) is available in Figure S3. The selected slice is the one next to the one centered on the needle. Temperature error up to 10°C is clearly visible,

Figure 5 compares the dosimetry performance with the proposed OF algorithms on the same respiratory‐gated acquisition (G#3). Again, the first and second rows (Figure 5a,b) show the magnitude images and a zoomed view. The accumulated thermal dose map (Figure 5c) displays a similar pattern but some variations can be noticed. Using the conventional OF method, the mask of estimated lesions is smaller by 1–3 voxels in diameter which corresponds to 2.5–7.5 mm. The time to thermal dose threshold map (Figure 5d) indicates that close to the tip, the cell necrosis threshold is reached in less than 12 dynamic acquisitions while in the border zone, up to 75 dynamic acquisitions were required. The difference in time to reach the accumulated thermal dose threshold map (Figure 5e) indicates that the conventional OF creates a delay (in orange) or an advance (in blue) of 20 dynamic acquisitions (∼40 s) in lesion size estimation. Accumulated thermal dose evolution in time in a ROI of 5 × 5 pixels was plotted in the right panel (Figure 5f) and illustrates the bias introduced by both tested methods versus the gold standard (in gold). To illustrate the reproducibility and reliability of the method, one additional case (G#5) are available in Figure S4 corresponds to the dosimetry computation of Figure S3.

**FIGURE 6:**
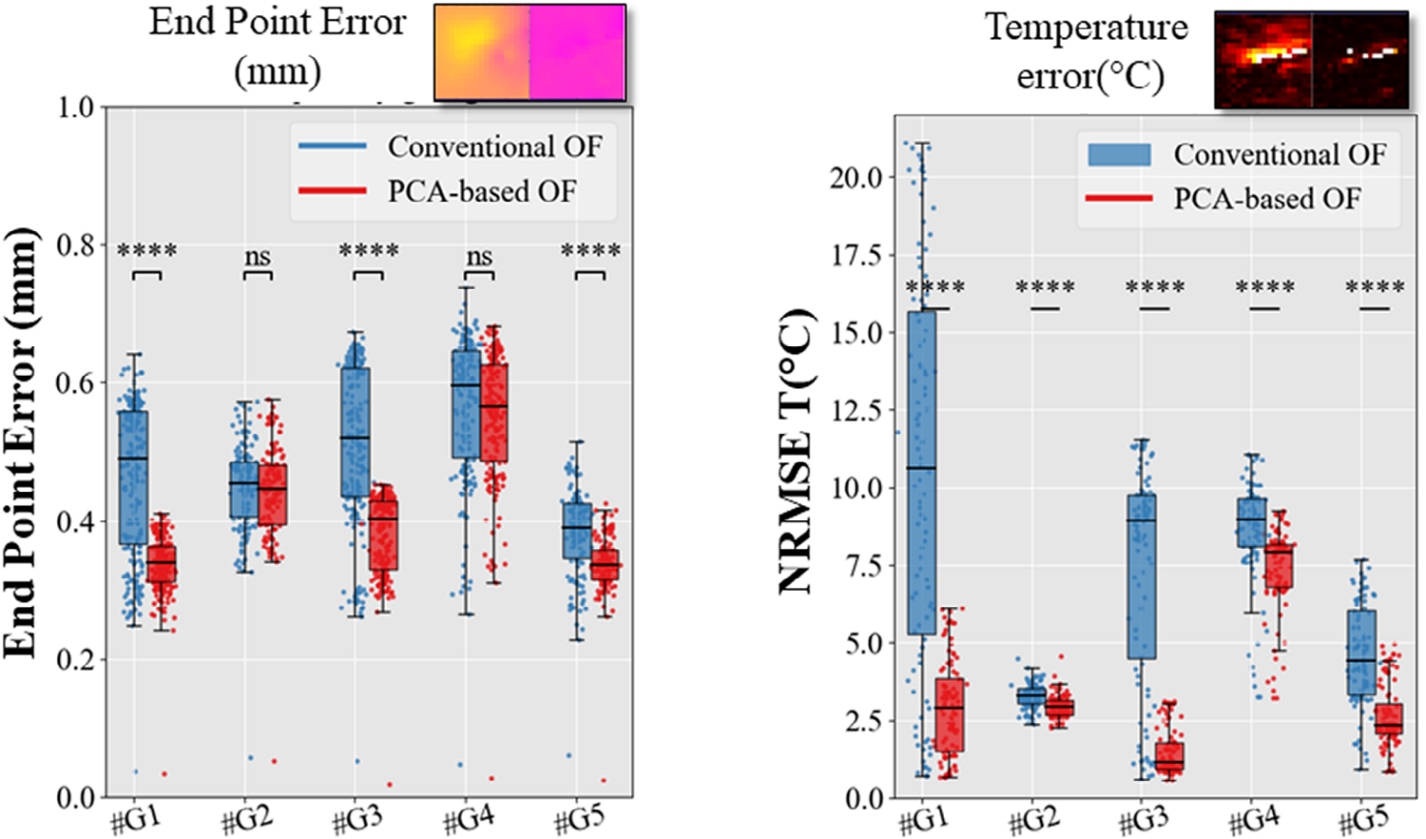
Comparison of motion estimation and thermometry performance. The box and whisker of the End Point Error (mm) and the NRMSE of temperature (T°C) are plotted in the left and right panels respectively. The *Conventional OF* (in blue) and the *PCA‐based OF* (in red) methods were compared to the *Gold Standard* approach. For both, the metrics were computed over 100 dynamic acquisitions taken during the heating period. NRMSE, normalized root mean square error; OF, optical flow.

A quantitative report of thermometry performance is presented in Figure 6 for all gated acquisitions. Again, the temperature NRMSE was found up to 20°C for the conventional OF method. A statistically significant decrease in averaged EE is observed for the PCA‐based OF with values close to 2.5°C, except for case G#4.

**FIGURE 7:**
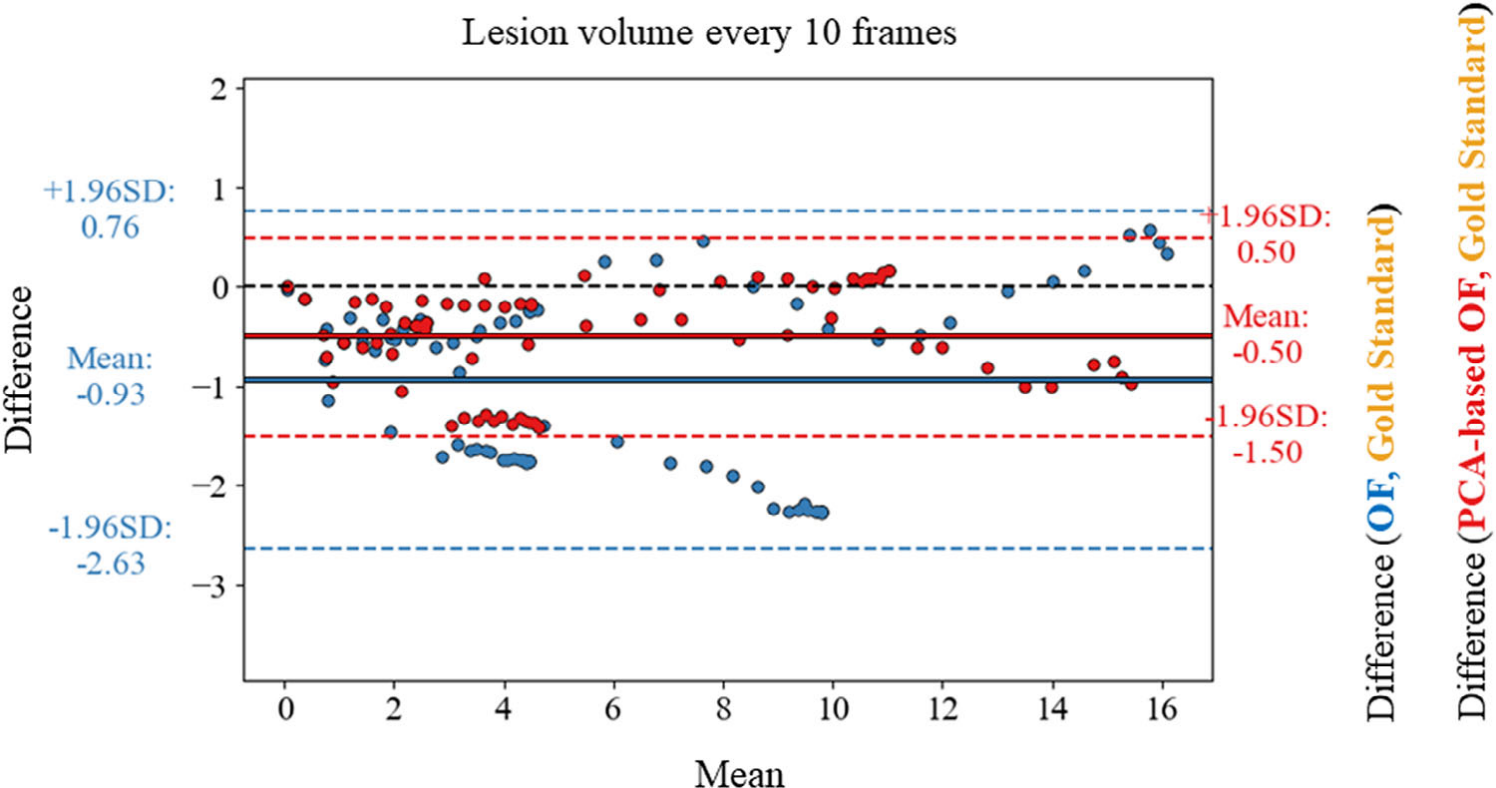
Evaluation of the lesion size estimation for the motion correction algorithms versus the gold standard approach. The resulting lesion size estimation in (cm^3^) is compared using Bland–Altman plots using the *Conventional OF* (in blue) and the *PCA‐based OF (in red) algorithms*. For each, the bias (solid lines) and limits of agreement (dotted lines) are indicated. A positive bias indicates an overestimation of the lesion size by the motion correction algorithms. A negative bias indicates an underestimation of the lesion size by the motion correction algorithms. OF, optical flow.

Figure 7 compares the lesion size estimation performance through the procedure. Each color indicates a comparison between the estimated volume using the gold standard versus the conventional OF (in blue) or PCA‐based OF (in red). An underestimation of the lesion size was found by both OF algorithms. The PCA‐based OF reported a bias of 0.5 cm^3^ with the 95% confidence interval below 2 cm^3^ (0.5 cm^3^/−1.5 cm^3^) while the conventional OF reported a bias of 0.93 cm^3^ and a greater dispersion (0.8 cm^3^/−2.6 cm^3^).

**FIGURE 8:**
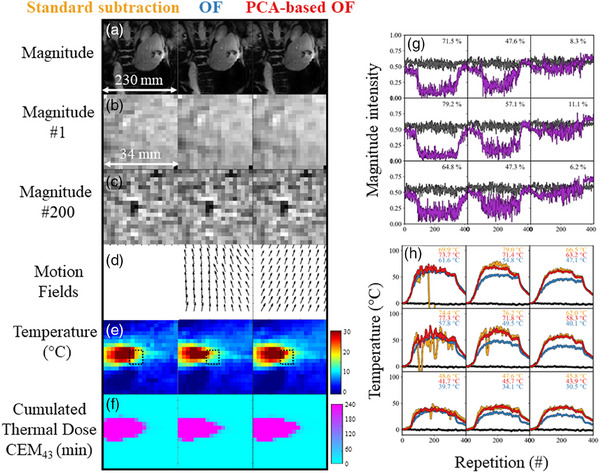
Case study of a non‐gated acquisition for comparing the thermometry and dosimetry performance with the proposed methods. Left panel: The first column corresponds to the approach without motion correction *(standard subtraction)*, the second to the *Conventional OF* (in blue), and the last one to the *PCA‐based OF (in red)*. Magnitude images (a) and a zoomed view (b) are shown close to the tip of the needle. Estimated motion field (c) after regridding (for a correct visualization). Lastly, temperature and accumulated thermal dose are shown. Right panel: Magnitude and temperature evolution as a function of dynamic acquisition is shown in a 3 × 3 kernel of voxels. Magnitude evolution at the vicinity of the needle and far from the needle is in purple and black respectively. The temperature is plotted using the color code introduced above. The black lines are another 3 × 3 kernel of voxel located far from the needle extremity. OF, optical flow.

*Fixed frequency acquisition results*. Figure 8 compares the thermometry performance with the proposed OF algorithms on a non‐gated acquisition (FF#6). The first, second and third columns (Figure 8a–c) show the magnitude images and a zoomed view at *t* = 0 s and *t* = 400 s. In this example, the image plane is oriented perpendicularly to the MW needle. The conventional OF estimated, at dynamic acquisition #200 (*t* = 400 s), a higher vector field (Figure 8d) than the PCA‐based OF. Magnitude signal and temperature evolution in time in a ROI of 3 × 3 pixels was plotted in the right panel (Figure 8g,h). A net decrease in magnitude signal (up to 71%) is visible (top left voxel). The temperature estimation (Figure 8e–h) without motion correction (in gold) led to strong fluctuations or loss of temperature measurement (around dynamic acquisition #180, top left voxel). The conventional OF algorithm recovers a stable measurement at the cost of a bias ranging from 10°C to 25°C. The proposed PCA‐based OF recovers both a stable and precise temperature measurement without bias.

To illustrate the reproducibility and reliability of the method, three additional cases (two for FF#6 and FF#2) are available in Figures S5–S7. The temperature estimation without motion correction and using standard subtraction (in gold) led to strong fluctuations or loss of temperature measurement which could be recovered by using the PCA‐based algorithm.

Figure S7 compares the lesion size estimation performance through the procedure on a non‐gated acquisition. An underestimation of the *Conventional OF* versus the *PCA‐based OF* algorithms was found with a bias of 0.47 cm^3^.

## 4 DISCUSSION

The present study performed at 1.5T using a commercially available multi‐slice EPI sequence investigated the impact of a new 2D DIR workflow to reduce potential bias in MR‐temperature estimation related to changes in T1/T2 tissue properties during the heating period. The proposed acquisition includes 13–20 slices acquired in an interleaved pattern in coronal or sagittal orientation. Phase image quality in all cases was found excellent as shown in Figure S9, this criterion is a prerequisite for obtaining high‐quality temperature maps.

The study first checked the impact of the triggering method on the image quality. The acquisitions were done either using respiratory gating or at a constant update rate of 2 s. The intercorrelation coefficient is a simple and fast metric to calculate for quantifying the degree of similarity between two images. Here, it is calculated over the whole image and can therefore be influenced by any disturbance of the magnitude signal on the liver or outside. Nevertheless, it proved to be very robust in characterizing the regularity of movements and the stability of image positions throughout each experiment (Figure S1 and S2).

The computation of NRMSE of magnitude images (Figure 3) through the acquisition also confirmed the absence of residual movement during gated acquisitions. The validity of the “no motion hypothesis” during the gated acquisition allowed us to investigate and compare the proposed OF algorithms against a gold‐standard approach. While alternative gold‐standard devices (such as probes) are sometimes accessible in phantom or preclinical experiments, such an option cannot be envisioned in clinical practice.

We then progressively present the potential bias that is generated on motion field estimation by the conventional OF method. This bias propagated for all calculated metrics, whether it be the temperature (Figure 4) or the accumulated thermal dose, the time to reach the thermal dose threshold (Figure 5), or the lesion size (Figure 7).

As shown in Figure 4 and Figure S3, the bias is much more significant at the core of the ablation because temperatures and associated decrease in magnitude signal are higher than at the periphery of the heated area where it becomes insignificant or null. This bias therefore introduces a significant error ranging from a few degrees to 20°C−30°C on the maximum estimated temperature increase. The error can be positive or negative, even though most of the time an underestimate of the actual temperature is observed. An error of assessment on this criterion introduces a safety risk for the procedure. During liver tumor ablation, high‐power MW settings are used to overcome the heat sink effect of perfusion, resulting in high‐temperature increase (sometimes higher than 100°C). A careful monitoring of the temperature is therefore needed.

An error in the maximum temperature measured is also critical for all applications using a feedback control loop for automatic temperature regulation. Although the initial work on this subject is well known,^28^ these approaches still remain marginal in clinical practice, and are mainly coupled to protocols on static organs using HIFU energy.^29^, ^30^ Improving the safety of the liver tumor ablation procedure requires both precise monitoring of the procedure and optimization of the ablation parameters depending on tissue response. Implementing such an approach would address both of these issues.

The PCA‐based OF succeeded in recovering the expected temperature measurements where conventional OF failed. A net decrease (up to 15°C) in NRMSE is observed in Figure 6. One major advantage of the proposed approach is that there is no penalty since it corrects what does not work and offers the same level of accuracy in locations where the decrease in magnitude signal is small or null (Figures S4 or S7). In most cases, the calculated temperature measurements were sufficiently accurate to provide a complete characterization of the thermal field during ablation (Figure 4 or Figure S4).

One part of the work sets out to show the reader how an error in the estimation of the vector field can ultimately impact the thermal dose, and therefore the estimation of the final ablated volume from thermal dose images. This error can also be reflected in the time required to reach the necrosis threshold. In this case, the bias on lesion size is not present at the core of the ablation, as the heating times and the energy sent are sufficient to reach the thermal damage threshold for both algorithms. Surprisingly, the bias still acts at the periphery of the ablation, with an underestimate of the temperature leading to an underestimate of the ablation volume. This intuition is confirmed in the five cases presented in the Bland–Altman graph comparing the two methods, where a greater underestimate (∼ 1 cm^3^) is found with the conventional algorithm. It is important to note here that the statistics and measurements are carried out on the ROI of size 19 × 19 x ∼7 slices centered on the tip of the needle for visualization purposes. A second reason is to keep the percentage of voxels affected by the drop in magnitude signal not too low. The aim of the quantification is therefore not to cover the entire volume of ablation performed nor to characterize the clinical result.

The second part of the study applied all approaches in another datasets acquired at a fixed frequency of 0.5 Hz without respiratory gating. The motion was relatively minimal as the patients were under general anesthesia. Typically, the images in the case FF#1 are mostly static but other cases present visible motion of the organs between consecutive images. Such impacts have been quantified by the NRMSE of magnitude intensity (Figure 3) and the intercorrelation coefficient (Figure S2). The computation of the PRF method therefore needed two corrections, one for freezing the motion between consecutive images, a second for correcting for magnetic susceptibility artifacts due to breathing. The two OF algorithms were applied and as a result, we observed a significant decrease in NRMSE of magnitude intensity (Figure 3), indicating that voxel misregistration through the acquisition was equally corrected by both algorithms.

A first case study with up to 80% signal decrease of magnitude signal is presented. Temperature estimation was lost or fluctuated in the absence of motion correction (*standard subtraction*) but was recovered by both OF algorithms. A clear bias was visible between both algorithms with an underestimation of 10°C to 25°C. In this specific case, the *standard subtraction* approach is readable and it was easy to conclude that the proposed PCA‐based OF did recover the correct temperature measurement. On the contrary, the presence of movement makes it difficult, if not impossible, to read *standard subtraction* temperature data in Figure S8 were recovered by the proposed OF algorithms. The new algorithm created no penalty where the decrease in magnitude signal is small or null (Figure S7). Again, in most cases, the calculated temperature measurements were sufficiently accurate to provide a complete characterization of the thermal field during ablation (Figure 8 or Figure S7). Lastly, as previously reported in Figure 7 for gated acquisition, a bias in lesion size estimation of approximately 0.5 cm^3^ was also reported between the two OF methods for fixed‐frequency acquisitions.

## 5 LIMITATIONS

Temperature rise induces changes in the tissues MRI properties, resulting in a drop in signal magnitude. However, this is not the only phenomenon that can produce such an effect. During high‐power MW ablation, the tissue temperature might reach locally the boiling point. The presence of air bubbles due to evaporation previously reported by^31^, ^32^ will create a bulk magnetic susceptibility artifact visible in Figure 8 in magnitude and temperature images. While not demonstrated here, the PCA‐based OF will help in minimizing potential bias induced by the presence of bubbles. Nevertheless, the correction of such artifacts^33^, ^34^, ^35^ was not investigated in this work.

The presence of spontaneous motions like a strong contraction of the liver happened in case FF#7 during the heating and were identified by the intercorrelation coefficient metrics. When analyzing the images of Fig S1C, movements are present both in the image plane and out of the plane. In this specific case, the use of 2D slice‐to‐slice OF algorithms is obsolete and requires a 3D approach.^36^ Such a scenario will strongly impact both the registration and the motion‐induced susceptibility correction and associated temperature estimation. The correction of such artifacts was not investigated in this work.

Clinical perspectives require in‐line integration of the algorithm in the Gadgetron framework. The second step is the validation of the computational time and associated latency. A CPU computation time, reported in the previous study,^21^ of 25 ms per image was found. At a fixed frequency of 0.5 Hz even with 20 slices (25 ms x 20 = 500 ms), the existing version would be therefore suitable for real‐time processing in the context of liver tumor ablation, without significant lag between acquisition and temperature display.

## 6 CONCLUSION

Motion field estimation using OF algorithm can be significantly affected by local variation of signal intensity on magnitude images associated with local tissue heating. A dedicated deformable image algorithm “PCA‐based” was designed and evaluated in a clinical setting in motion‐free datasets and apply then with ones with motion. An accurate assessment of the vector field and temperature was achieved, enabling the size of the lesions to be better quantified. The approach was evaluated using single‐shot EPI, a widely available sequence used in diffusion, perfusion, and fMRI that provides high‐quality phase images of the liver in clinical practice and offers wide spatial coverage (6 cm depth) while maintaining a fast acquisition rate (20 slices / 2 s) to monitor large lesions formation.

## CONFLICT OF INTEREST STATEMENT

Valéry Ozenne and Bruno Quesson are co‐funders and shareholders of Certis Therapeutics. Pierre Bour, Thibaut Faller, and Manon Desclides are employees of Certis Therapeutics.

## Supporting information



Supporting Information
