# Supplementary material for: Evaluation of a deformable image registration algorithm for image‐guided thermal ablation of liver tumors on clinically acquired MR‐temperature maps
Source: Med Phys. 2024 Nov 23;52(2):722–36. doi: 10.1002/mp.17526 (PMC11788246; doi:10.1002/mp.17526)
Supplement: Supplementary file 1 — Supporting Information [file MP-52-722-s001.docx]

Supplementary figures


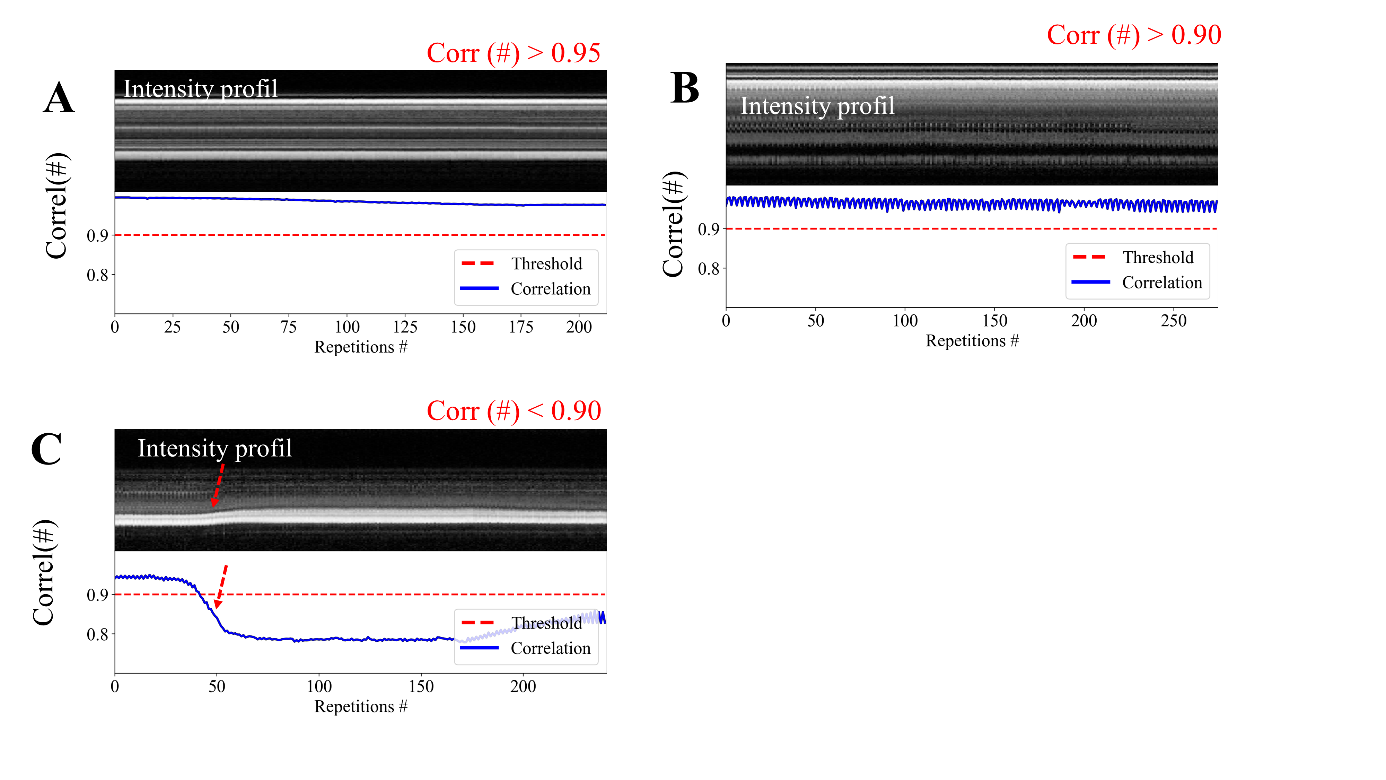


**Supplementary Figure 1. Representative case of physiological motion during the procedure and detection.** For each panel, the intensity profiles in head foot direction are plotted (upper part) jointly with the intercorrelation coefficient (downer part) computed between the current frame and the reference frame and plotted as a function of dynamic acquisition. The red dotted line indicates an arbitrary threshold describing the similarity of the images throughout the acquisition. Case A was a respiratory-gated acquisition. Case B was a non-gated acquisition. Case C was a non-gated acquisition where significant movement or contraction of the liver occurred during ablation.


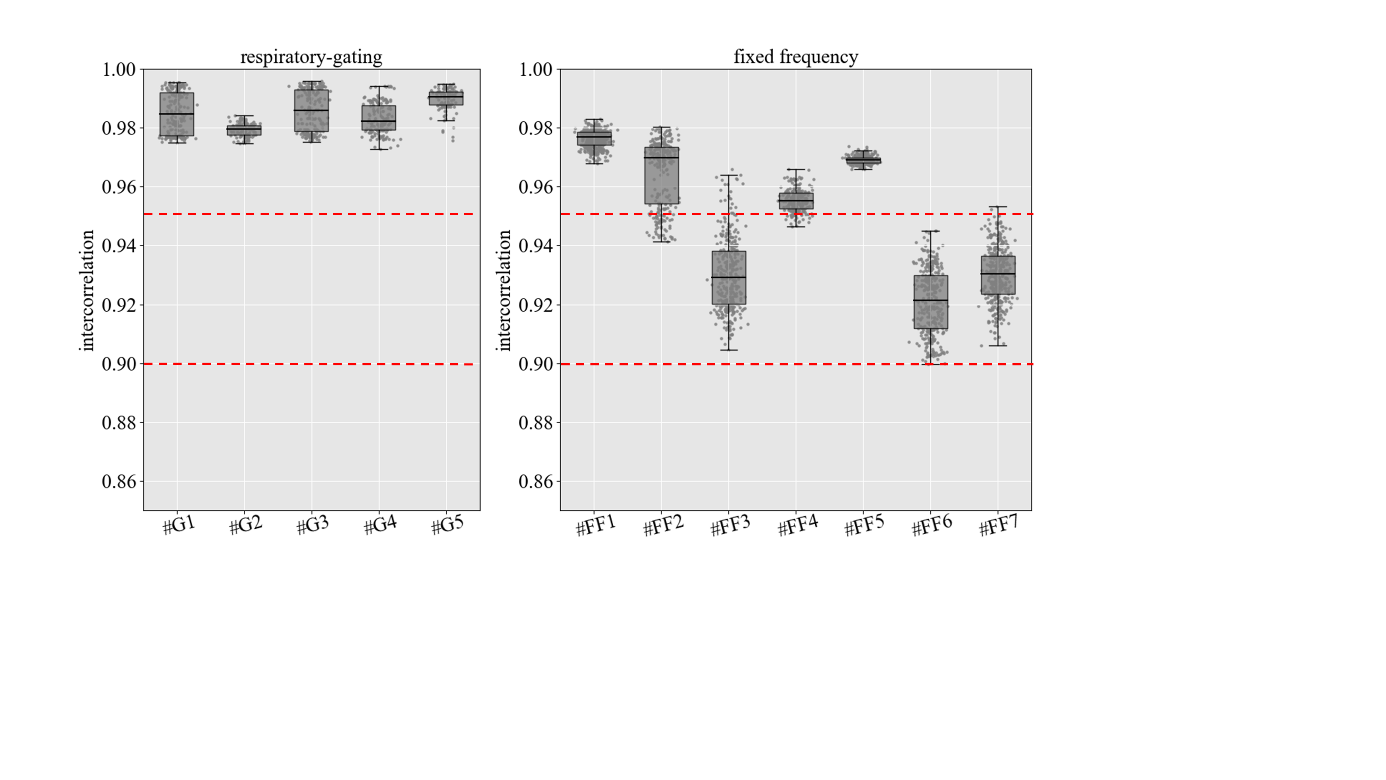


**Supplementary Figure 2. Comparison of image similarity quality using the intercorrelation** **coefficient** for respiratory gated (left) and non-gated (right) acquisitions. The box and whisker plots use the intercorrelation coefficients calculated on all the images in the acquisition. The red dotted lines indicate two arbitrary thresholds describing the similarity of images throughout the acquisition.


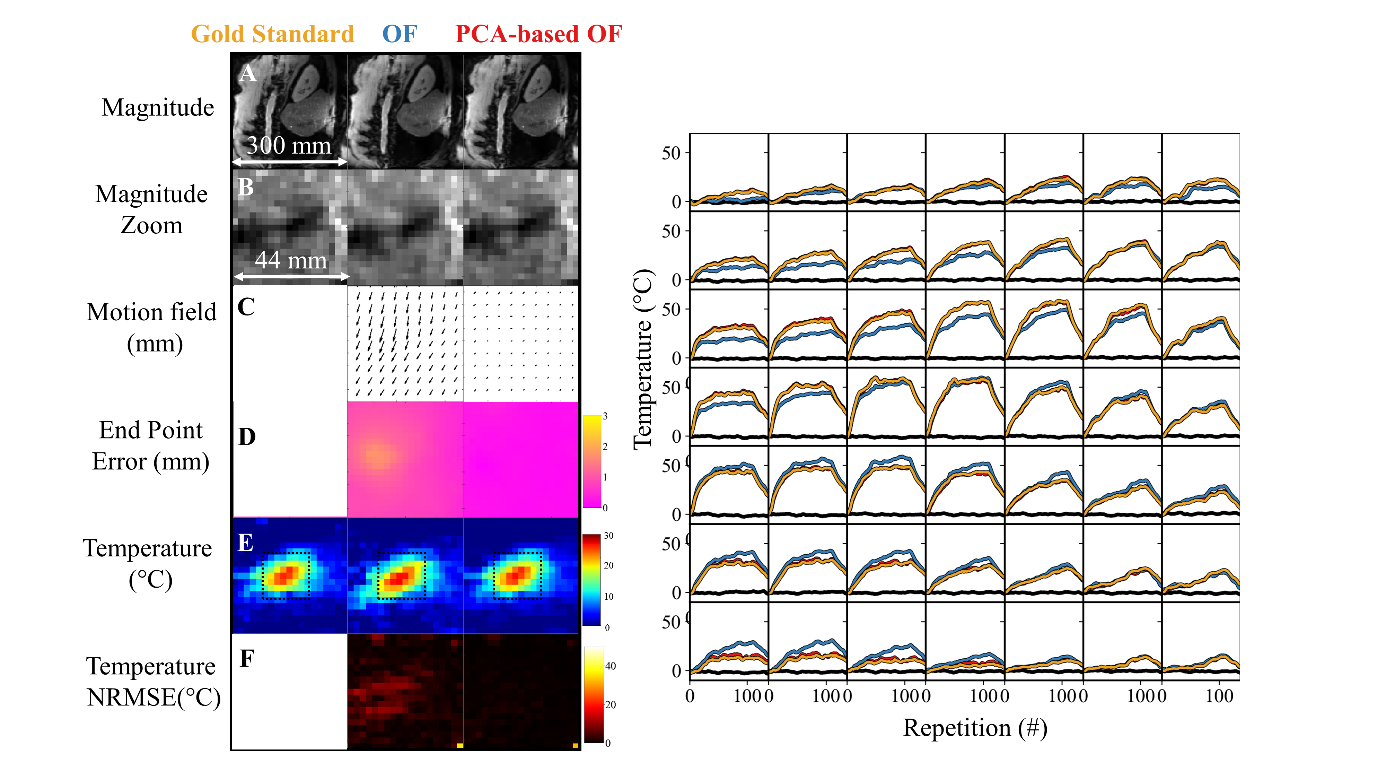


**Supplementary Figure 3.** The legend of figure 4 applies.


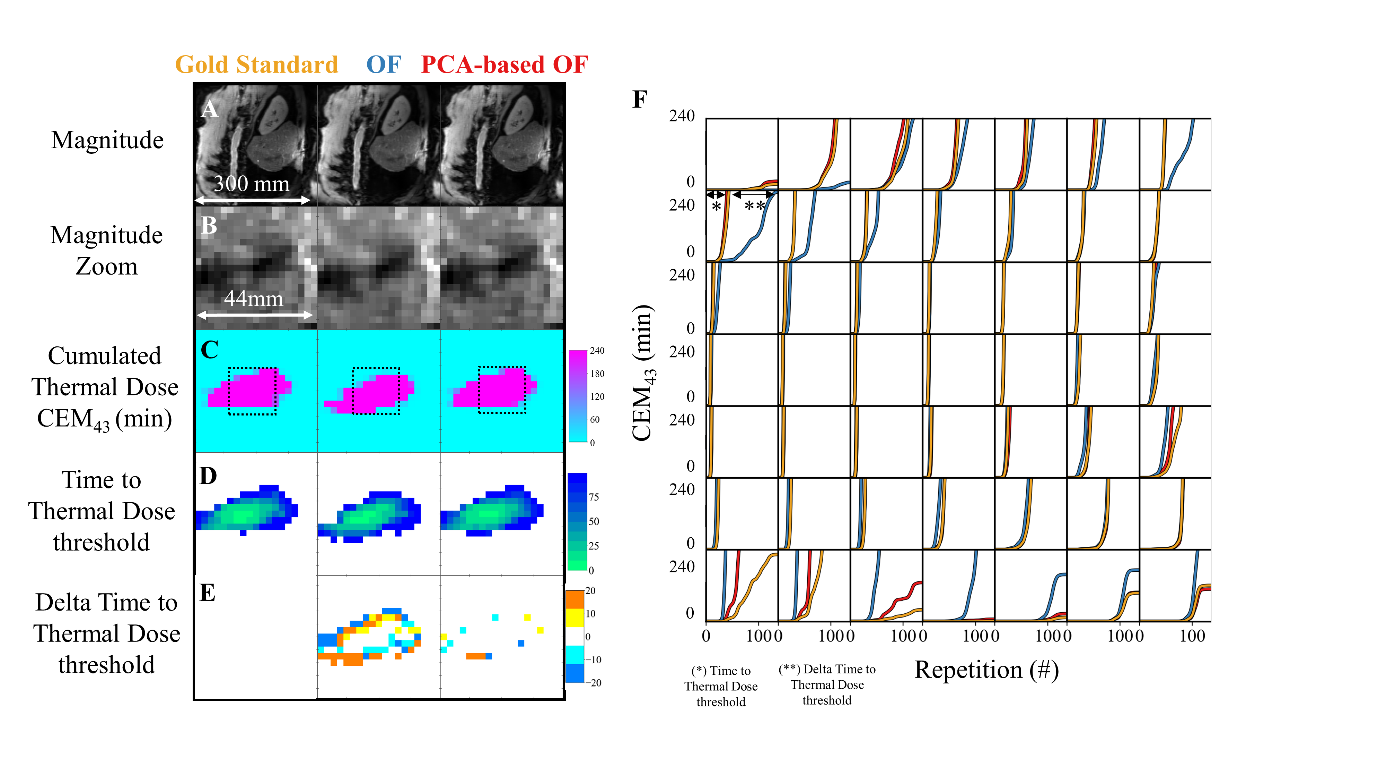


**Supplementary Figure 4.** The legend of figure 5 applies.


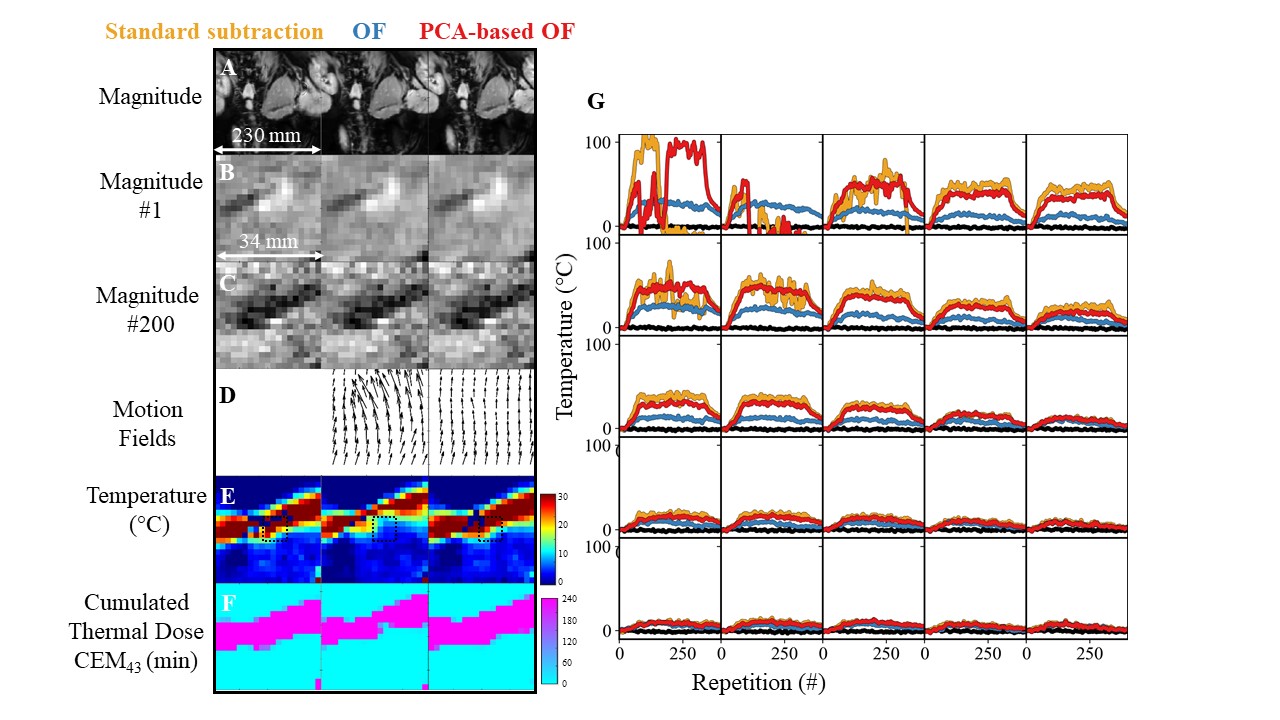


**Supplementary Figure 5.** The legend of figure 8 applies. The selected slice is the one next to the one centered on the needle. Strong fluctuations are visible at the vicinity of the device but are minored by the OF. There is a clear difference between the estimation results of the two algorithms proposed.

**
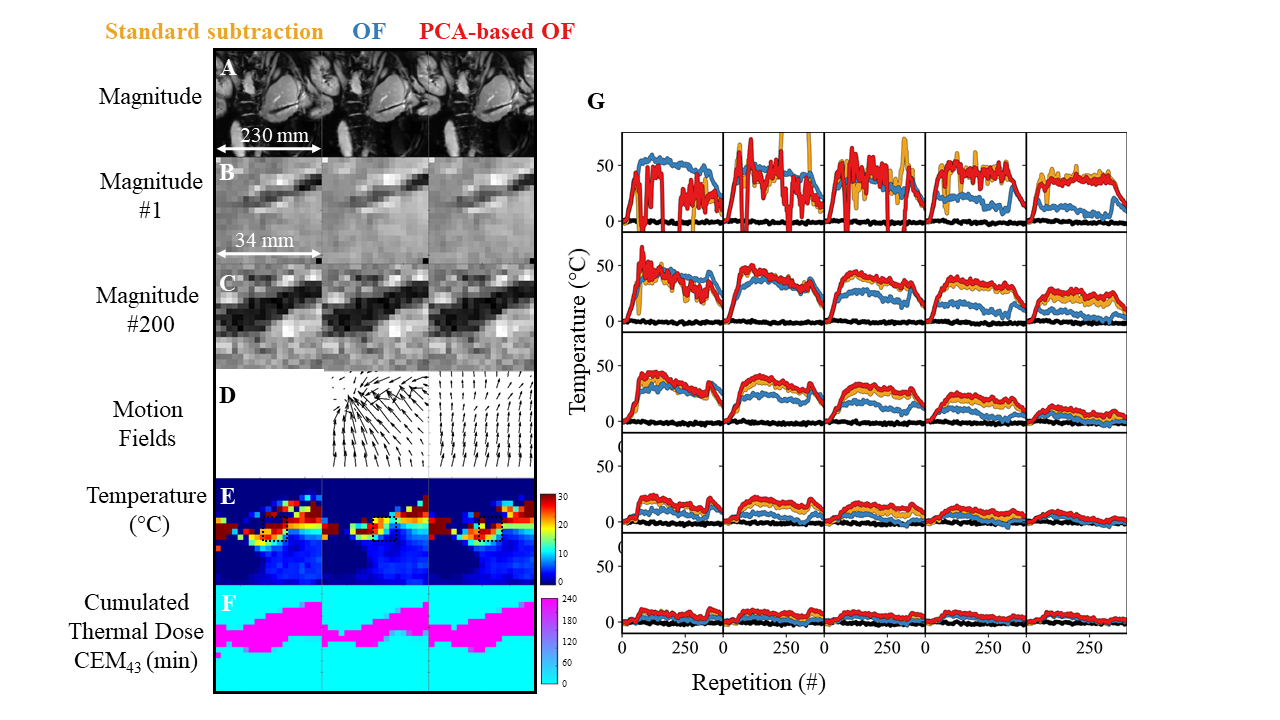
**

**Supplementary Figure 6.** The selected slice corresponds to the case shown in Fig. 2 and is centered on the needle. The legend of figure 8 applies. Strong fluctuations are visible at the vicinity of the device but are minored by the OF. There is a clear difference between the estimation results of the two algorithms proposed. Temperature error up to 20°C.

**
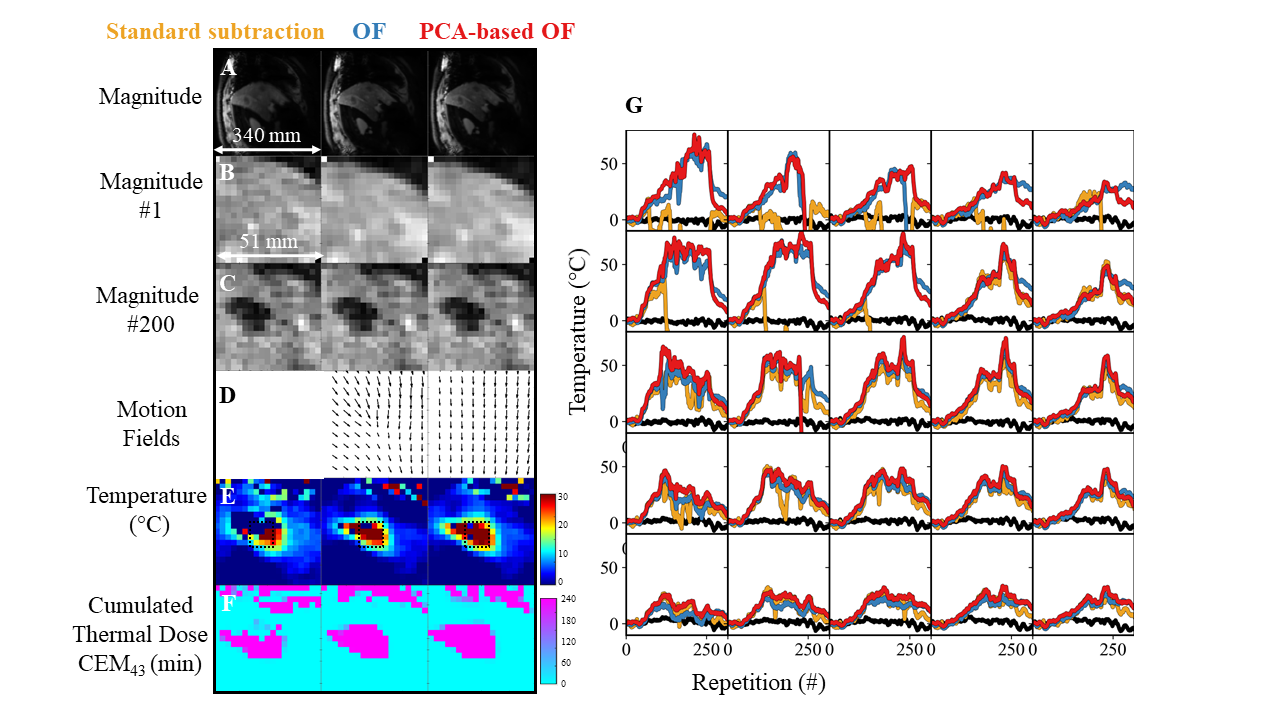
**

**Supplementary Figure 7.** The legend of figure 8 applies. The image plane is oriented obliquely to the MW needle


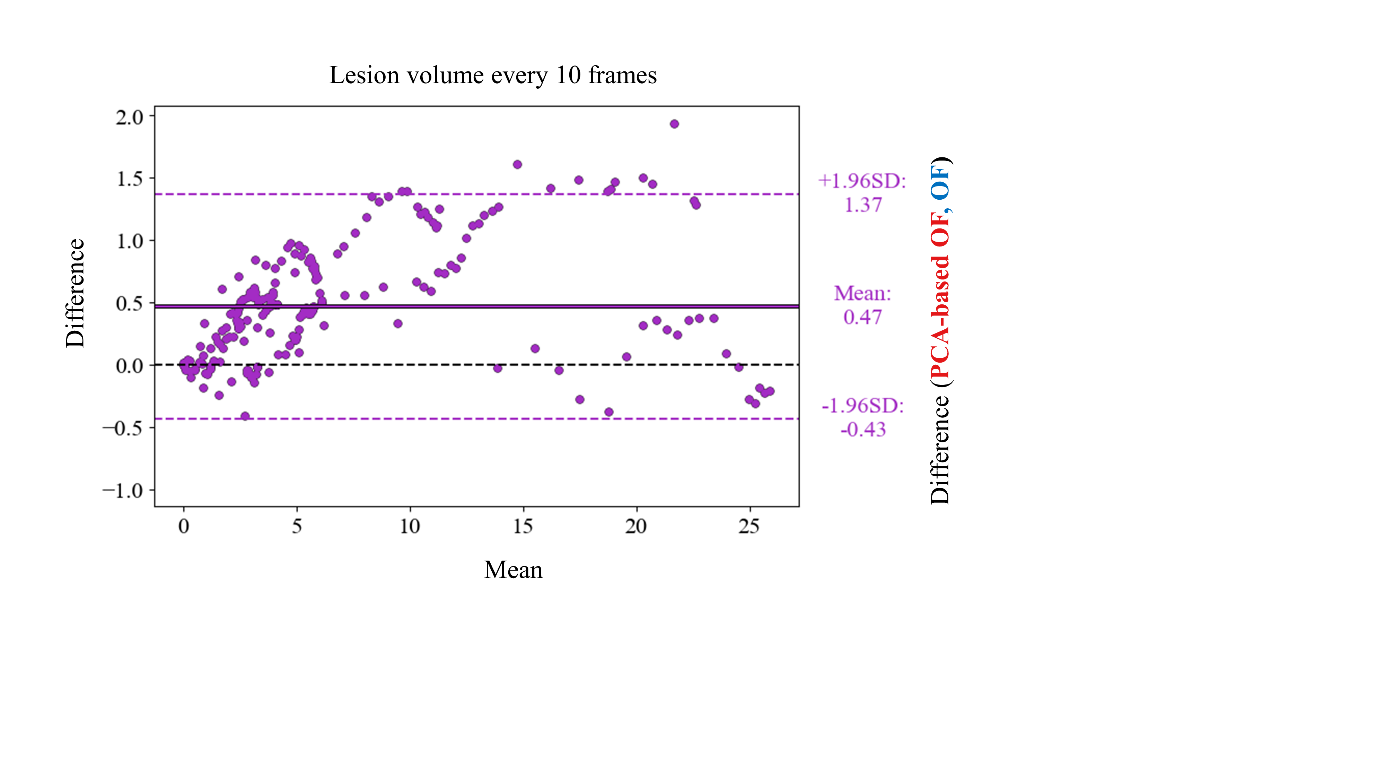


**Supplementary Figure 8: Evaluation of the lesion size estimation between the two OF algorithms.** The resulting lesion size estimation in (cm^3^) is compared using Bland Altman plots. The bias (solid lines) and limits of agreement (dotted lines) are indicated. A positive bias indicates an underestimation of the *Conventional OF* versus the *PCA-based OF* algorithms and backwards.

**
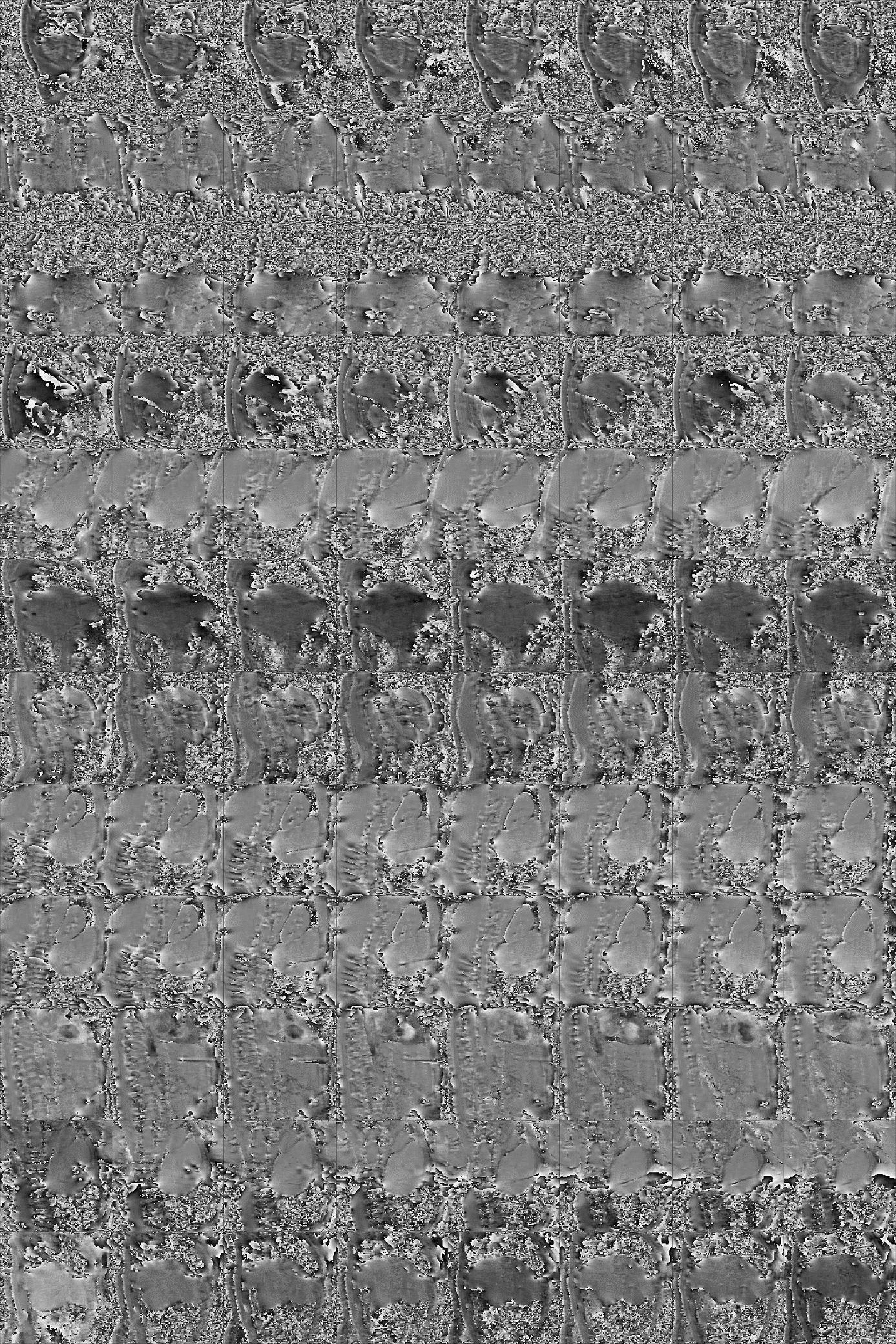
**

**Supplementary Figure 9. Phase images for the cases.** Slices #3 to #10 are shown. Excellent phase image quality was found for all cases
